# Influences on willingness to use vaginal or oral HIV PrEP during pregnancy and breastfeeding in Africa: the multisite MAMMA study

**DOI:** 10.1002/jia2.25536

**Published:** 2020-06-10

**Authors:** Ariane van der Straten, Julia H Ryan, Krishnaveni Reddy, Juliane Etima, Frank Taulo, Prisca Mutero, Jamilah Taylor, Jeanna Piper, Petina Musara, the MTN‐041/MAMMA Study Team

**Affiliations:** ^1^ Women’s Global Health Imperative (WGHI) RTI International Berkeley CA USA; ^2^ Center for AIDS Prevention Studies (CAPS) University of California San Francisco San Francisco CA USA; ^3^ University of Zimbabwe College of Health Sciences Clinical Trials Research Centre Harare Zimbabwe; ^4^ Wits Reproductive Health and HIV Institute (Wits RHI) University of the Witwatersrand Johannesburg South Africa; ^5^ Makerere University – Johns Hopkins University Research Collaboration Kampala Uganda; ^6^ Johns Hopkins Project‐College of Medicine University of Malawi Blantyre Malawi; ^7^ FHI 360 Durham NC USA; ^8^ DAIDS NIH/NIAID Bethesda MD USA

**Keywords:** vaginal dapivirine ring, Truvada pills, HIV, safety, stigma, socio‐ecological framework, acceptability

## Abstract

**Introduction:**

Women in sub‐Saharan Africa spend a substantial portion of their reproductive lives pregnant and/or breastfeeding (P/BF), yet they have limited options to prevent HIV during these maternal stages. In preparation for phase 3b prevention trials in P/BF women, we explored attitudes about using a vaginal ring or oral pills for pre‐exposure prophylaxis (PrEP), perceptions of HIV risk during P/BF and key influences on future PrEP use.

**Methods:**

In 2018, we conducted 16 single‐sex focus group discussions (FGDs) with community‐ and clinic‐recruited HIV‐uninfected women, currently or recently P/BF, aged 18 to 40, and men with (currently or recently) P/BF partners, aged 18+. Participants completed a behavioural questionnaire, viewed an educational video and handled prototype placebo products. FGDs were conducted in local languages and transcribed, coded and analysed, using a socio‐ecological framework, for key influences on willingness to use products, HIV risk perceptions and opinions on product attributes.

**Results:**

Of the 128 participants (65 women, 63 men) 75% lived with their partner and 84% had a child. Women reported the most important influencers when P/BF were partners, and all stated that health decisions when P/BF are typically made jointly (e.g. medication use; ante/postnatal and baby care). There was consensus that P/BF women are at high risk for HIV, primarily because of their partner’s infidelities, and new prevention options were welcomed. Participants valued multiple options and stated that woman’s personal preference would be key to product choice. Anticipated concerns about products included risk of miscarriage, impact on infant development, complications during delivery and adequate production or taste of breastmilk. Specific perceived disadvantages emerged for the ring (e.g. vaginal discomfort, difficulty inserting/removing) and for pills (e.g. nausea/vomiting) that may be exacerbated during pregnancy. Health care providers’ (HCPs) knowledge and approval of product use during P/BF was needed to mitigate anticipated fears.

**Conclusions:**

Participants perceived pregnancy and breastfeeding as high HIV risk periods and valued new prevention options. HIV protection of the mother‐child dyad, safety of the baby, and ultimately, health of the family were paramount. Endorsement by HCPs and support from partners were key to future product acceptance. Participants recommended involving partners and HCPs in sensitization efforts for future trials.

## 1 INTRODUCTION

High fertility rates in sub‐Saharan Africa translate to women spending substantial time in their reproductive years (12% to 29%) either pregnant or breastfeeding [1, 2]. Pregnant and/or breastfeeding (P/BF) women are at high risk of acquiring and perinatally transmitting HIV. Previous studies suggest that two of the leading causes of HIV acquisition by P/BF women are physiologic changes during gestation that may increase susceptibility and spousal infidelity that results in male partners contracting HIV and then exposing their wives to the virus [3, 4]. Few prevention options are available to P/BF women, highlighting the urgent need for safe and effective methods during these periods [5].

Current recommendations for HIV prevention during pregnancy and breastfeeding include HIV testing and linkage to care, condom promotion, sexually transmitted infection screening and treatment, and partner‐related strategies [6]. Daily oral Truvada™ is approved for HIV Pre‐Exposure Prophylaxis (PrEP) in all populations at risk [7] and is safe and effective in P/BF women who are at risk of HIV infection [8, 9]; however, women’s access to oral PrEP services remains limited [10]. The dapivirine vaginal ring (a microbicidal PrEP approach) prevented HIV in phase 3 trials [11, 12], and open‐label phase 3b trials confirmed its safety and effectiveness in nonpregnant women [13, 14], with regulatory approval process underway.

P/BF women are rarely studied in microbicide/PrEP trials because of concerns about potential harm to the foetus and baby. Thus, there is little safety data for pregnancy and lactation periods [15, 16, 17, 18, 19]. Fewer studies explored acceptability of topical and oral PrEP during these periods [20, 21]. Oral PrEP and the vaginal ring provide women with tools to protect themselves from infection without requiring negotiations with their partners, as is the case with condoms. In preparation for open‐label phase 3b PrEP trials in P/BF women, we explored perceptions of HIV risk and attitudes about using a monthly ring or daily pills among recently or currently P/BF women and partners of P/BF women in sub‐Saharan Africa. This paper focuses on opinions about these products and key influences on willingness to use them during pregnancy and breastfeeding.

## 2 METHODS

### 2.1 Study design

MTN‐041/MAMMA (Microbicide/PrEP Acceptability among Mothers and Male Partners in Africa), a multisite study in Blantyre, Malawi; Johannesburg, South Africa; Kampala, Uganda; and Chitungwiza, Zimbabwe, was conducted between May and November 2018. Sixteen single‐sex focus group discussions (FGDs) were conducted across all sites, with individuals independently recruited into the following groups: (a) self‐reported HIV‐uninfected women aged 18 to 40 who were currently or recently (in the past two years) P/BF; or (b) men aged 18+ with female partners who were currently or recently (in the past two years) P/BF. Participants were recruited from various community settings (e.g. street outreach, community advisory board members, word of mouth, construction sites (men only)), antenatal and postnatal clinics (women only)); 129 were screened and eligible and 128 joined the study.

### 2.2 Study products

Placebo pills for daily oral PrEP (identical in appearance to Truvada, Gilead Sciences, Foster City, CA, USA) and a silicone elastomer placebo ring (identical in appearance to dapivirine vaginal ring, International Partnership for Microbicides, Silver Spring, MD, USA) were presented for handling during FGDs.

### 2.3 Procedures

**Figure 1:**
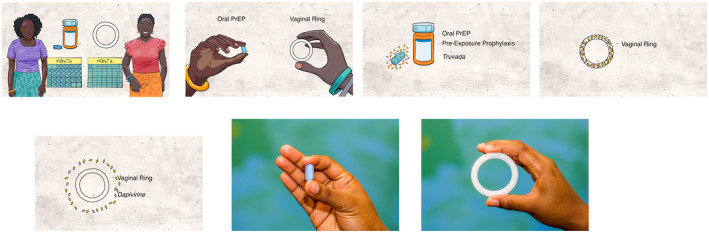
Screenshots from educational video and demonstration placebo study products. The four‐minute educational video (https://vimeo.com/262813431/dd19ece7dc) was presented at midpoint into the FGD, just prior to moving to the section of the discussion on new prevention products. It described briefly the HIV prevention landscape and the two study products (daily oral pills and monthly vaginal ring), including mechanism of action and how each is to be used. Placebo products (as pictured) that were identical looking to the active dosage forms were passed along during the FGDs so participants could touch and feel both. FGDs, focus group discussions.

Demographic and behavioural information were collected through interviewer‐administered questionnaires translated into local languages (Chichewa in Malawi, Zulu or English in South Africa, Luganda in Uganda and Shona in Zimbabwe). The decision‐making dominance subscale was adapted from Pulerwitz *et al*. [22]. The food insecurity item was based on the Household Food Insecurity Access Scale [23]. FGDs were conducted in these languages using semi‐structured guides by gender‐matched trained local social scientists. All site staff were fluent in the local language of their country, and FGD participants were required to speak and understand the language in order to participate. Topics discussed included HIV risk perceptions, health‐related decision making, key influencers, and interest in HIV prevention methods while P/BF. Just prior to discussing new HIV prevention options, participants viewed a brief educational video and handled prototype placebo products (Figure 1). FGDs were audio recorded, transcribed and translated into English. Interviewers completed a summary report after each FGD for rapid thematic analysis.

### 2.4 Data analysis

Demographic and behavioural data on decision making, HIV prevention methods awareness/use and key influencers are presented descriptively in Tables 1 and 3. Differences by geographical site for women’s responses were calculated using Fisher’s exact tests (Table 3).

**Figure 2:**
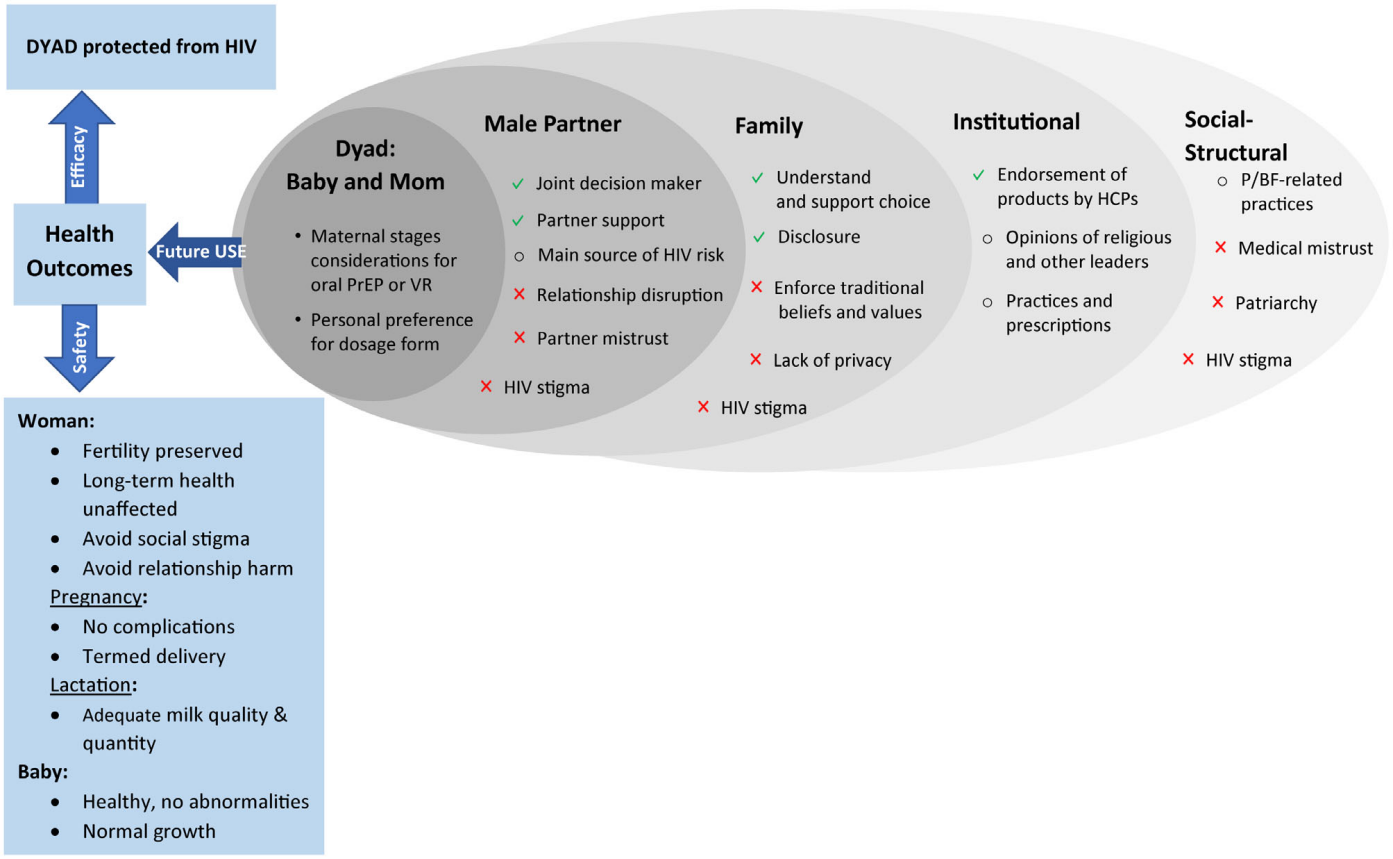
Socio‐ecological spheres of influences on future use of HIV prevention products during p/BF periods. Sphere of influence on future product use included the mother and baby dyad, the spouse (or male partner or father of the baby) at the closest interpersonal level, followed by family members (mostly grandmother of the baby, siblings and other family members). Institutionally, important stakeholder included health care providers (doctors, nurses, etc.) and religious leaders. At the socio‐structural level, salient influences included pregnant or breastfeeding‐related permissible or forbidden practices, community rumours that fuelled HIV stigma (influencing all levels from socio‐structural to their partner’s opinion of the products), fear of health innovations, such as PrEP, as a manifestation of general medical mistrust, and patriarchal gender norms favouring the sexual double standard. Salient health outcomes aligned with dyadic protection for efficacy, and with safety, for those exposed to PrEP and VR (the woman and the baby), as well as with the maternal stages of pregnancy and lactation. 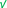 = perceived facilitators; 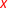 = perceived barriers; 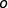 = other topics of influence acting either as perceived facilitators or barriers. Pregnant and/or breastfeeding.

All sites research staff attended several analysis workshops and participated in a rapid preliminary analysis of the data they had collected [24]. The findings generated during the workshops directly informed the iterative development of the codebook, which in turn was used to systematically analyse all qualitative data. Transcripts were coded by four coders (Dedoose software, v7.0.23) using the codebook that followed a socio‐ecological framework (Figure 2). An acceptable level of intercoder reliability was set and maintained at approximately 80% agreement for 10 key codes representative of the main topics of interest. The analysis team met weekly to discuss coding questions, issues and emerging themes. Coded data reports were further summarized thematically into analytical memos that were reviewed by site teams. Pseudonyms are used in presenting quotes to protect the identity of participants.

All participants provided written informed consent prior to participation. The study protocol was approved by the Western Institutional Review Board and by local IRBs at each of the study sites and was overseen by the regulatory infrastructure of the U.S. National Institutes of Health and the Microbicide Trials Network.

## 3 Results

Overall, 128 participants joined a single‐sex FGD; 65 were P/BF women, and 63 were (independently recruited) male partners of P/BF women (Table 1). The median number of participants across all FGDs was 8.5 (range 5 to 10). Most (84%) had children and 41% were currently pregnant (or had a partner currently pregnant); 74% of women had breastfed. Household composition was strikingly different in South Africa compared to other locations: overall, 75% lived with their partner and 62% with their children, although in South Africa a third or less reported so. Overall, a fifth lived with other adult relatives (parents, grandparents, siblings, in‐laws), yet over half of the South African participants reported so. Most participants had used male condoms previously; however, less than half were aware of the vaginal ring or oral PrEP, with 3 women and 2 men reporting previous oral PrEP use.

**Table 1:** Characteristics of sample by sex, country (alphabetically ordered) and overall

Variable	P/BF women (N = 65)	Men (N = 63)	Malawi (N = 31)	South Africa (N = 27)	Uganda (N = 37)	Zimbabwe (N = 33)	Total (N = 128)
Mean age (range)	27.1 (19 to 40)	30.6 (19 to 54)	28.5(19 to 53)	30.2(22 to 49)	29.9(19 to 54)	26.8(19 to 45)	28.8(19 to 54)
Secondary education completed	33 (51%)	35 (56%)	12 (39%)	19 (70%)	13 (35%)	24 (73%)	68 (53%)
Earns an income	27 (42%)	48 (76%)	22 (71%)	4 (15%)	29 (78%)	20 (61%)	75 (59%)
Food scarcity^a^	21 (32%)	16 (25%)	7 (23%)	8 (30%)	9 (24%)	13 (39%)	37 (29%)
Christian religion	63 (97%)	53 (84%)	29 (94%)	24 (89%)	31 (84%)	32 (97%)	116 (91%)
Married or living with partner	50 (77%)	51 (81%)	29 (94%)	8 (30%)	32 (87%)	32 (97%)	101 (79%)
Household composition/living with							
Spouse/partner	47 (72%)	49 (78%)	28 (90%)	7 (26%)	30 (81%)	31 (94%)	96 (75%)
Other adult relative^b^	13 (20%)	13 (21%)	4 (13%)	14 (52%)	3 (8%)	5 (15%)	26 (20%)
Children	44 (68%)	35 (56%)	24 (77%)	9 (33%)	22 (60%)	24 (73%)	79 (62%)
Partner might be having sex with someone else^c^							
≥Agree	24 (37%)	3 (5%)	5 (16%)	7 (26%)	9 (24%)	6 (18%)	27 (21%)
Pregnancy and breastfeeding history							
Currently pregnant (self or spouse)	32 (49%)	20 (32%)	11 (36%)	9 (33%)	19 (51%)	13 (39%)	52 (41%)
Mean pregnancies (range)	2.4 (1 to 7)	NA	2.5 (1 to 5)	1.9 (1 to 3)	3.1 (1 to 7)	2.3 (1 to 5)	–
Ever breastfed^d^	48 (74%)	NA	12 (80%)	10 (67%)	15 (83%)	11 (65%)	–
Decision‐making score, during pregnancy and breastfeeding^e^ (1: father, 2: both equally, 3: mother)							
Pregnancy mean score (median)	2.4 (2.4)	2.2 (2.2)	2.2 (2.2)	2.6 (2.5)	2.3 (2.4)	2.2 (2.3)	2.3 (2.3)
Breastfeeding mean score (median)	2.4 (2.4)	2.2 (2.2)	2.3 (2.3)	2.5 (2.6)	2.2 (2.2)	2.3 (2.3)	2.3 (2.3)
Awareness of HIV prevention methods							
Male condoms	65 (100%)	63 (100%)	31 (100%)	27 (100%)	37 (100%)	33 (100%)	128 (100%)
Oral PrEP	29 (45%)	28 (44%)	9 (29%)	12 (44%)	21 (57%)	15 (46%)	57 (44%)
Vaginal ring	23 (35%)	21 (33%)	8 (26%)	10 (37%)	20 (54%)	6 (18%)	44 (34%)
Ever used HIV prevention methods							
Male condoms	51 (79%)	60 (95%)	27 (87%)	26 (96%)	33 (89%)	25 (76%)	111 (87%)
Oral PrEP	3 (5%)	2 (3%)	0 (0%)	3 (11%)	1 (3%)	1 (3%)	5 (4%)
Vaginal ring	0 (0%)	0 (0%)	0 (0%)	0 (0%)	0 (0%)	0 (0%)	0 (0%)

P/BF, Pregnant and/or breastfeeding; PrEP, pre‐exposure prophylaxis.

^a^ Participant worry in past four weeks that s/he would not have enough food (4‐point Likert scale). Combines strongly agree and agree

^b^ All adult relations combined. Includes sibling, parent, and grandparent (from most common to least common)

^c^ Four response options: strongly disagree to strongly agree; agree and strongly agree combined for presentation

^d^ Only asked to women in the sample

^e^ Response to five questions “who has more say when making decisions about the following [topics]?” (during pregnancy: her medication and vitamin use, antenatal care and HIV testing, where she delivers, her diet and nutrition, her use of traditional medicines; during breastfeeding: her medication and vitamin use, postnatal care and HIV testing, where the baby goes for well‐baby visits, her diet and nutrition, her use traditional medicines). Score range 1 to 3, 1: male partner/father has more say, 2: both equally, 3: P/BF woman/mother has more say.

### 3.1 HIV risk perception and motivation for new prevention product use

Pregnancy and breastfeeding were overwhelmingly recognized as high HIV risk periods for women. P/BF women were perceived to be biologically weaker. Nevertheless, the main perceived driver of women’s risk was their male partner’s sexual behaviour. As shown in Table 1, 40% of women agreed with the statement “my partner has other sexual partners.” In contrast, only 5% of men agreed with the statement that their female partner has other sexual partners. Given consensus in FGDs that men put their P/BF female partners at risk and husband’s general unwillingness to use condoms with their pregnant wives, participants concurred that new prevention methods are needed during these vulnerable times:

I should say that men are not being faithful. It is good if a woman puts on a ring as a man, if not faithful, and maybe the woman is pregnant and the man goes to have a girlfriend [..]It will be good if the woman has the ring, if the man had contracted a disease….then the woman can be protected. [Collins, 19, man, Blantyre]

Women echoed this view:

I think most people will accept [products] because we are many mothers and we are the ones who are in most need of staying healthy. I think women will accept to use it because they do not trust men. [Sarah, 28, woman, Kampala]

Multiple spheres of influences on P/B women willingness to use new HIV prevention technologies in the future were identified. As illustrated in Figure 2, a key motivation for future use of the pills or ring was dyadic protection, and ultimately for mothers to protect their babies from HIV:

The thing that comes to my mind is that I will give birth to my children without any worries of infecting them with HIV. [Pamela, 26, woman, Kampala].

Other spheres of influences to future use (discussed in more detail below) involved male partners, who were seen as the primary source of support, but also the main source of HIV risk for P/BF women; family and household members whose understanding of these novel products could support prevention choice for P/BF women; trusted medical, religious and cultural leaders whose endorsement of the products would be necessary for future uptake; and finally, social‐structural context that may primarily act as a barrier for adoption of these technologies, but that could be modified by social and institutional actors.

### 3.2 Attitudes about the ring and pills

Participants welcomed the ring and pills for HIV prevention during pregnancy and breastfeeding:

I think it would be good for me [man] to hear that these products like the vaginal ring here, is important for the woman to prevent her from getting HIV when it is inserted. Because I love my wife, I would be excited to know that she is safe and protected. [Sheriff, 24, man, Chitungwiza]

Women wanted protection for themselves, for their baby and thus, for the whole family:

…if the woman has made a choice to use the pill or the ring you are protecting your life and also making your family healthy. [Deborah, 34, woman, Blantyre]

Overall, women liked having options and emphasized personal preference and autonomy:

You are not going to choose for them [..]Women will choose what they want to use, and at what time they want to use these products [Makhosi, 40, woman, Johannesburg]

Both the mother’s and baby’s health were key when weighing product’s efficacy (i.e. remaining HIV free) and discussing safety (Figure 2). General thoughts about the ring and pills emerged, along with concerns specific to use during pregnancy and lactation. For example, forgetfulness with pill taking was thought to be exacerbated during pregnancy and breastfeeding. Table 2 summarizes anticipated product benefits and concerns – including possible side effects – as expressed in FGDs, with exemplary quotes.

**Table 2:** Anticipated benefits and concerns with ring and pill use, overall and during pregnancy and breastfeeding periods, with representative quotes

Vaginal ring	
Benefit	
Long acting/ease of use	*it is like a tampon, so I don’t think it can interfere. Even the tampon is worse because it gets full and you can feel it is full but the vaginal ring stays there for a month so I don t think it can interfere with your life*. [Lisa, 27, woman, Johannesburg
Discreet/private	*People will say, “Ah, what are these pills for?” and they can actually say that you have HIV. The ring is discreet. If husbands are not cooperative, women can just use it as long as it will not be felt during sex* [Tanatswa, 25, woman, Chitungwiza]
Local drug exposure	*Since it was explained that the ring protects only the vaginal part so I don’t think it’s going to have an effect on the child while breastfeeding because the medicine doesn’t go up to the breasts it only settles on the vagina*. [Ngwanenyana, 26, woman, Johannesburg]
Concern	
Interference with sex	*What I worry about is for it (ring) to make the woman less lubricated. It can even torture me psychologically because I know that there is something in the woman’s vagina. I may fail to enjoy sex with this woman having the ring*. [Emma, 23, man, Uganda]
Vaginal insertion taboo	*It is not allowed for a pregnant woman to be inserting the ring in the vagina because when a woman is pregnant there are many problems or I should say that so many happenings in the same vagina… I should say that she cannot be busy having time to take care of the ring…*[Chikondi, 30, man, Blantyre]
Vagina enlarged/overloaded	*Already as I progress in pregnancy the vagina will be enlarging and with this* [Ring] *now, what will the sex partners say? They will not enjoy sex*. [Jane, 30, woman, Chitungwiza]
Baby entangled or injured during delivery	*What if I am at home and have labour pains and deliver there, it [vagina ring] will strangle the baby, how will the baby come out? the baby must come through that tube, yes it will interfere. It will block the baby, cause of infections and all that, and hurt the baby because you would have forgotten because labour pain will be heavy on you*. [Nonhlanhla, 34, woman, Johannesburg]
Misperceived as abortion tool^a^	*They [HCPs] will ask you if you want to abort the baby, that’s another thing, they will ask what is it, do you want to abort?* [India, 31, woman, Johannesburg]
Exacerbates physical discomfort of pregnancy	*Because you are uncomfortable already about everything, your body changes more often, the baby is moving and so on, so your thoughts are filled with what if I am going to labor and maybe I push this thing [the ring] hard and it disturbs the baby, you have got all those things in your head so yeah you won’t be comfortable throughout*. [Apple, 24, woman, Johannesburg]
Problem inserting/removing ring	*What I fear about it…I feel it is big and hard. Can I really insert such a thing? You are telling us that we remove it ourselves but how do I do that?* [Vanessa, 23, Woman, Kampala]
May affect milk production	*My question is about breastfeeding women, won’t it [vaginal ring] have some side effects like…you know some family planning methods affect breastfeeding mothers and they can’t have enough breast milk…so my question is, won’t breastfeeding mothers lose breast milk?* [Suzan, 29, woman, Uganda]

Oral pills	
Benefit	
Pill familiarity	*I don’t think it will be difficult for us to take this pill, or it will not be difficult for me because I use oral contraceptive pills* (Jane, 30, woman, Chitungwiza)
Protection from multiple sexual routes^a^	*These pills can help for both oral and vaginal sex because there are those who prefer oral sex to vaginal sex*. [Grey, 26, woman, Johannesburg]
Tested and approved	*As something that was tested and seen that we can use it, I do not think it will be difficult when we are taught and we understand. I do not foresee any problems for us to use it because it was already tested by doctors and they saw it fit for us to use it.”* [Tendai, 37, woman, Chitungwiza]
Oral medication may benefit the fetus or baby	*I am siding with [Makhosi] because we do take pills while we are pregnant like the vitamins and they tell you it is for blood or high blood pressure, so this one will be treated the same, for high blood [pressure] to protect the child and make the baby grow, so there will be no worries*. [Nonhlanhla, 34, woman, Johannesburg]
Concern	
Pills are for sick people	*The worry I have is that I hear people saying that when a person takes drugs when she is not sick, it causes poison in her body. I don’t know if it is true or not. Now, won’t it [PrEP] cause our loved ones (wives) to get poison in their bodies and then we start seeking treatment for poison?* [Farouk, 54, man, Uganda]
Mistrust in relationships	*I doubt they [men] would agree and want their partners to use it. For them it would be questionable, how do you use such a product knowing that it’s just me and you… it would be like undermining their manhood because, [..] you can always go outside [cheat]* [Mastermind, 29, man, Johannesburg]
Oral pills taboo	*…yes it is good that the drugs [pills] will protect from HIV, but they may bring some undesirable side effects while you are pregnant; as it is said that when one is pregnant, she should not be taking drugs*.[Lucy, 29, woman, Blantyre]
Forgetfulness with daily dosing	*Taking that tablet every day during pregnancy helps to prevent HIV but you can forget to take it and have engage into sex with a man and when you test you find yourself infected with HIV*. [Sharon, 19, woman, Kampala]
Poor health effects of bitter drugs	*People believe that when a woman is pregnant taking bitter things affect the baby that can result in the death of the unborn baby and these bitter things can be prohibited in the community*. [Unite, 28, woman, Blantyre]
Exacerbate nausea and other symptoms	*I would [starting on PrEP] after two months when the baby has grown otherwise during those early days pregnancies are so delicate. That is when a woman vomits a lot and tablets also have their side effects…I think it should be started after four months*.[Esther, 22, woman, Uganda]
Risk of miscarriages during first trimester	*I would think that it is better to start taking it when at least the womb is about three months. I think tablets might affect it and end up with a miscarriage if you take them at one week or a month*. [Suzan, 29, woman, Uganda]
Potent drug may disable baby	*The first thing I think about is whether it won’t affect the baby because you have told us that we can take it during pregnancy but won’t it affect the baby and probably deliver when is disabled?* [Agatha, 21, woman, Uganda]
Compatibility with traditional medicine	*I think it is important to tell the traditional healer you are using the ring or pill, imagine she will give you that concoction to drink and it will take away the effectiveness of the pill* [India, 31, woman, Johannesburg]
Side effects and drug interaction with hormones	*As a pregnant mother, what first comes into my mind is the issue of side effects to me and the baby, because just like any other pill there are side effects. The other thing is, since my hormones are already tempered around with because of pregnancy, will the PrEP pill go down well with me?* [Tanya, 31, woman, Chitungwiza]
Increased appetite	*I think that tablet might increase a woman’s appetite yet she doesn’t have what to eat and she is pregnant*. [Barbra, 36, woman, Uganda]
Drying up milk	*In as much as I want to prevent myself from HIV, the other thing that comes into my mind is what is going to happen if they dry up my milk, yet I am supposed to do exclusive breast feeding in the first 6 months*. [Tanya, 31, woman, Chitungwiza]
Milk contamination	*I will be worried that maybe the breastmilk will be bitter*. [Mpho, 27, woman, Johannesburg]
HIV stigma	*After delivery, you go to your parents’ home to nurse the baby and there are pills that you need to take at a certain time [..].Always at 8 p.m. you must take it. That’s when they will gossip about you and suspect that you are positive, “why is she not telling us?” you see!* [Nonhlanhla, 34, woman, Johannesburg]

PrEP, pre‐exposure prophylaxis.

^a^ Minority view in Johannesburg.

Main safety considerations for P/BF women included avoiding long‐term adverse effects of the products related to fertility, reproductive tract infections and cancers. Participants also discussed relationship safety, including both preserving trust and minimizing interpersonal harm:

[If] a woman is pregnant… and taking these pills, for me it would be like she has another partner, or she doesn’t trust me. And I would ask myself… is it her trust in me or is it our health? Because if she is protecting like that, I would respect her for that but if we use them without communicating…I cannot continue with that relationship.” [Sipho, 30, man, Johannesburg]

Safety considerations for the baby focused on delivering at term and having a healthy child (e.g. normal growth without developmental abnormalities). The novelty of the ring and pills as PrEP strategies raised anxiety about their possible effect on the baby. Women especially expressed fear of miscarriages:

I heard that when you take those pills you get a miscarriage. Now, how sure am I that when I take it I will not get a miscarriage? I have seen someone before who started taking it and got a miscarriage. [Samantha, 30, woman, Kampala]

In Blantyre, taking a bitter tasting drug (as pills were perceived to be) was thought to possibly induce abortions, whereas in Johannesburg, bitterness was associated with drug potency (Table 2). Several participants noted that vaginal insertions during pregnancy were taboo and presented an obstacle to ring use:

We believe that inserting things in the vagina disrupts the baby and he can die while in the womb. That is why traditionally, inserting things in the vagina is not allowed especially when the woman is pregnant. [unidentified man, Kampala]

Some women said that pills may be difficult to take in early pregnancy, resulting in more severe pregnancy symptoms, such as nausea and vomiting. Also, taking medication during pregnancy is traditionally taboo, unless explicitly prescribed by a doctor. Hence, the ring was preferable to some for early pregnancy and for breastfeeding, as it presumably would not contaminate breastmilk with drugs that could harm the infant. However, during late pregnancy, the ring could be challenging to insert/remove, may exacerbate physical discomfort by overburdening the vagina, and interfere with a smooth delivery of the baby, if not removed in time.

Anticipated concerns with taking pills during breastfeeding focused on drying up the milk or spoiling its taste. Additionally, fear of exposing the unborn or breastfeeding baby to the preventative drugs was commonly mentioned.

### 3.3 Key influencers

Women reported that their partner was the most influential person in general decision‐making during pregnancy or breastfeeding (Table 3). The P/BF woman’s mother, doctor and other family members were also influential. Both women and men stated that health decisions when P/BF are typically made jointly (e.g. medication use; ante/postnatal and baby care; Table 1). FGDs corroborated survey data and reinforced partners as the dominant influencers in a P/BF woman’s decision to use prevention products. Some men stated that a woman’s mind and body are weaker during pregnancy and represent a time when the partner’s role as provider and protector is particularly important. Accordingly, men’s role included giving permission to their P/BF partners for using an HIV prevention method.

**Table 3:** Women’s reported key influencers during pregnancy and breastfeeding, overall and by country

Key influencers during pregnancy	Malawi (N = 15)	South Africa (N = 15)	Uganda (N = 18)	Zimbabwe (N = 17)	Fisher’s Exact *p*‐value	Total (N = 65)
The father of your baby	9 (60%)	4 (27%)	11 (61%)	15 (88%)	0.001	39 (60%)
Your mother^a^	3 (20%)	6 (40%)	3 (17%)	0 (0%)		12 (18%)
Your clinician	3 (20%)	0 (0%)	3 (17%)	2 (12%)		8 (12%)
Other, specify^b^	0 (0%)	3 (20%)	0 (0%)	0 (0%)		3 (5%)
No response	0 (0%)	1 (7%)	1 (6%)	0 (0%)		2 (3%)
No one	0 (0%)	1 (7%)	0 (0%)	0 (0%)		1 (2%)

Key influencers during breastfeeding	Malawi (N = 12)	South Africa (N = 10)	Uganda (N = 15)	Zimbabwe (N = 11)	Fisher’s Exact *p*‐value	Total (N = 48)
The father of your baby	11 (92%)	2 (20%)	8 (53%)	9 (82%)	0.007	30 (63%)
Your mother	1 (8%)	4 (40%)	3 (20%)	1 (9%)		9 (19%)
Your clinician	0 (0%)	2 (20%)	4 (27%)	0 (0%)		6 (13%)
Other, specify^c^	0 (0%)	2 (20%)	0 (0%)	0 (0%)		2 (4%)
No one	0 (0%)	0 (0%)	0 (0%)	1 (9%)		1 (2%)

^a^ Two noted mother‐in‐law;

^b^ aunt (2), sister (1)

^c^ aunt (1), sister (1).
At church they say that the head of the family is the man, so a woman may want [to use] but if the man doesn’t it cannot be possible. So, the first one to have the decision is the man. [Obama, 35, man, Blantyre]

Women also expressed needing partner support to keep peace in the family:

Because even the pill or the ring can cause conflicts in the home. So, they are things you need to discuss and agree first before using them. Women must not think of using this thing [Ring] without agreeing with their partners. [Jane, 30, woman, Chitungwiza]

Because of the mother–daughter bond and going through the same maternal experiences previously, grandmothers were highlighted as key influencers of P/BF women too, especially in Johannesburg. Regarding encouraging pill or ring use, some mentioned that grandmothers may side with the elders and promote more traditional approaches to prevent HIV because they may not understand new products. Furthermore, they could fuel HIV‐related stigmatization, pushing some women to favour the more discreet product:

… I think the ring is better. These pills are tricky, if your family members start to see you taking these pills every day they will not understand you. Even when you try explaining that it is to prevent HIV some of our parents might take long to understand or might even think that you are on ART. [Tsitsi, 32, woman, Chitungwiza]

At the institutional level, health care providers’ (HCPs) are trusted medical experts. Their approval and endorsement were fundamental in building credibility for using these novel products during P/BF and encouraging their uptake.

I trust a doctor, like a medical doctor…this one [medical] has been trained, and when s/he gives you something you will know that it is safe. [Kgomotso, 33, man, Johannesburg]

Other institutional‐level influencers such as chiefs, traditional providers or religious leaders, emerged during FGDs as potential PrEP influencers who could build trust in communities.

At the socio‐structural level, willingness to use (or support use of) PrEP was influenced by two major negative issues: HIV stigma and medical mistrust. Concerns with stigmatization of P/BF woman was frequent, especially if taking pills.

With the pill, some of us we have a belief that once we see someone taking pills, we quickly think that the person is sick [HIV positive]…so in her community, she will be stigmatized that she is HIV positive and is on antiretroviral therapy… [Pizza, 28, man, Chitungwiza]

Other reactions to new PrEP products highlighted ongoing fears of racial discrimination and general mistrust of medical research:

Some women will decline to use it, saying that “the Whites have come to kill us just like it is for family planning methods.” When they give birth to babies who have some disabilities, they say that it was due to family planning methods. So, I think some women will say that it is about Whites trying to get involved in everything. [Angel, 24, woman, Kampala]

### 3.4 Willingness to Use (women) or Recommend Use (men)

Overall, participants had favourable attitudes towards new HIV prevention options for P/BF women, as condoms are typically not acceptable in marital relationships. The following recommendations were provided to facilitate future adoption and use by P/BF women and support by key influencers:

1. Gain providers approval and endorsement:
  I think it should be the clinic or hospital that tell you whether you should take it or not while breastfeeding. There might be chances that it affects [the baby] or not, so I think it should be the professionals who tell you whether it is okay for you take it or not [Grey, 26, woman, Johannesburg]
2. Target efforts to gain partners’ support, which would include men‐focused educational campaigns, engaging them as advocates and providing couple education and counselling.
  Men can understand but also become willing that their wives can take part in the study. […] He can be one who educate and campaign to men so that men can understand better. [Chikondi, 30, man, Blantyre]
3. Increase general awareness and educate future users, partners and community members to better understand and trust PrEP.
  It is hard for people to accept something if they only hear about it for the first time. When something is new, it might be impossible for people to accept that. So likewise, for these methods that we have just learnt today, there is need to be discussing on them frequently so that people should accept them little by little. [Favour, 29, woman, Blantyre]

Participants believed that acceptance was linked to comprehensive explanation of how the products worked, including potential side effects.

Participants specifically emphasized the value of personal testimonials by including product ambassadors as spokespersons who can “*speak to people and explain what she is experiencing [..]It is just like when you come to a hospital for delivery and you have fears but when you see someone delivering it can encourage you*.” [Aidah, 22, woman, Kampala].

## 4 DISCUSSION

We explored opinions of and interest in using the vaginal ring and daily oral pills for HIV prevention among P/BF women and partners of P/BF women in sub‐Saharan Africa. Women and men’s views were generally aligned, indicating that these are periods of high vulnerability for women, primarily because of men’s infidelities, highlighting the need for new options to protect women, the mother–baby dyad, and the overall health of the family. Similar findings were reported in a previous study of PrEP use during pregnancy in Kenya in which participants perceived male partner behaviour as the primary driver of HIV risk during pregnancy and expressed that PrEP may serve as a potential risk mitigation strategy [25]. Providing choice was empowering, and women’s personal preference was a main reason to select one product or the other. Nevertheless, general and maternal stage‐specific concerns were discussed for each product. Some attributes of oral dosing were anticipated to be problematic in early pregnancy (e.g. nausea and vomiting) and breastfeeding (e.g. changed production or taste of milk). Similar concerns about PrEP side effects and safety during pregnancy were reported among HIV‐uninfected Kenyan women in serodiscordant relationships [26]. The bulkiness and vaginal placement of the ring raised concerns about insertion/removal in late pregnancy or possible interference during delivery.

We used a socio‐ecological framework to organize the levels and types of influences on future PrEP use. Women’s partners were portrayed as directly engaged in approving prevention decisions, highlighting men’s potential supportive and authoritative role [27, 28]. Husbands were simultaneously described as women’s protectors and controllers and main source of HIV risk [21, 29, 30]. Cohabitation status may affect the degree of men’s influence. For example, in Johannesburg, where over half of women lived with other adult relatives, partners were comparatively less influential; more often, women cited grandmothers as key influencers. Grandmothers may be underutilized stakeholders to encourage PrEP use; efforts to engage and educate them to support their daughters may prove fruitful [31, 32]. Health providers’ approval and endorsement were highlighted as critical (but perhaps not sufficient) to accept PrEP. HCPs were trusted and viewed as central to alleviating fear and enhancing credibility of PrEP, especially during pregnancy and breastfeeding when taboos around taking medication exist [21, 33].

From the socio‐structural through the partner level, the impact of HIV stigma dampened PrEP interest. In settings of high HIV prevalence, taking antiretrovirals for prevention – particularly daily pills – is viewed as stigmatizing because it can be a marker of HIV infection [34, 35, 36, 37]. General fear of health innovations and medical mistrust blended with racial concerns emerged in some FGDs. The parallel between PrEP and contraceptives to control African populations was mentioned, suggesting that concerns persist across settings and indications [38, 39].

The ring generated more questions than pills, perhaps because pills are a more familiar dosage form [40, 41]. In previous studies, this initial reluctance to use a vaginal device was overcome once the method was tried and users experienced its ease of use and discreetness [42]. The main product‐specific concerns were not specific to pregnancy and breastfeeding; nevertheless, some issues, such as the size of ring, painful placement, increased nausea or forgetting daily PrEP were anticipated to be exacerbated, particularly during pregnancy. As in other populations, the ring was favoured for its discretion, monthly duration and local drug exposure [43], and appeared more suitable during lactation or early pregnancy. Pills were described as preferable after the first trimester and considered highly potent because of the systemic route of delivery; however, concerns were raised about their impact on quality and quantity of breastmilk production. During pregnancy, both products were feared to be associated with miscarriage. Generally, taking medications and inserting vaginal products were proscribed in pregnancy, a reason why explicit endorsement from medical personnel was perceived as critical.

It is important to consider that novelty can invoke fear when introducing health innovations, particularly in P/BF women when avoiding harm to the growing baby is paramount. Reassurance from the medical establishment prior to widespread use or full regulatory approval will be important for future trial recruitment. Participants advised that communities require thorough education on both products to alleviate fear and recommended involving men and HCPs in sensitization efforts. Central messaging for women should focus on protecting babies from HIV as participants thought this would override other product‐related fears. Leveraging testimonials from product ambassadors was encouraged since social norms for PrEP do not yet exist. Because of PrEP’s novelty, new users may desire social proof from trusted individuals with more expertise in using these products.

There are several limitations to this study. First, we enrolled a convenience sample recruited for exploratory research, with no intention of generalizability. Our objectives were to elicit salient opinions and attitudes about the pills and ring to directly inform the development and implementation of future phase 3b trials [44, 45]. However, the multicountry sample adds strength to our findings that mostly were aligned across geographical settings. The community‐based recruitment, with mostly product‐naïve participants, may best reflect the views of end‐users overall, who have not had access to either products outside of a clinical trial setting. Finally, qualitative data analysis is interpretive. We involved all sites in analyses and maintained quality control and interpretation meetings throughout the analysis period to maximize consensus.

## 5 Conclusions

Oral PrEP and the vaginal ring are effective prevention methods with clear potential to reduce women’s disproportionate burden of HIV during their lifespan, including during pregnancy and breastfeeding [10, 46, 47]. Participants recognized the critical need for new options during these vulnerable times, and the importance of engaging multilevel influencers to encourage their adoption and use, as well as participation in future trials. One challenge will be to overcome initial concerns by thoroughly educating and supporting women and their loved ones, so that evidence can be generated to approve use of these novel life‐saving technologies during pregnancy and breastfeeding.

## Competing interests

Dr. van der Straten received non‐financial support from Gilead sciences outside the submitted work. The authors declare no other conflicts of interest.

## Authors’ contributions

AVS wrote the paper, served as the protocol chair of MTN‐041 and led the development and implementation of the study together with PM, her protocol co‐chair. JR led the analysis of qualitative data. KR, JE, FT, PM and JT were directly involved with study implementation and the acquisition of data. JP contributed to the study design and served as the Medical Officer for the study. All authors provided critical feedback and helped shape the research, analysis and manuscript.
